# Should radioiodine now be first line treatment for Graves’ disease?

**DOI:** 10.1186/s13044-020-00077-8

**Published:** 2020-03-09

**Authors:** Onyebuchi E. Okosieme, Peter N. Taylor, Colin M. Dayan

**Affiliations:** 1 Thyroid Research Group, School of Medicine, Cardiff University, Cardiff, UK; 2 Diabetes Department, Prince Charles Hospital, Cwm Taf University Health Board, Gurnos Estate, Merthyr Tydfil, CF47 9DT UK

**Keywords:** Graves’ disease, Hyperthyroidism, Radioiodine therapy, Thyroidectomy, Mortality, Major adverse cardiovascular events

## Abstract

**Background:**

Radioiodine represents a cost-effective treatment option for Graves’ disease. In the UK, it is traditionally reserved for patients who relapse after initial thionamide therapy. In a change from current practice, the new guidelines of the National Institute for Health and Care Excellence (NICE) recommends that radioiodine should now be first line therapy for Graves’ disease. However, the safety of radioiodine with respect to long-term mortality risk has been the subject of recent debate. This analysis examines evidence from treatment related mortality studies in hyperthyroidism and discusses their implications for future Graves’ disease treatment strategies.

**Main body:**

Some studies have suggested an excess mortality in radioiodine treated cohorts compared to the background population. In particular, a recent observational study reported a modest increase in cancer-related mortality in hyperthyroid patients exposed to radioiodine. The interpretation of these studies is however constrained by study designs that lacked thionamide control groups or information on thyroid status and so could not distinguish the effect of treatment from disease. Two studies have shown survival advantages of radioiodine over thionamide therapy, but these benefits were only seen when radioiodine was successful in controlling hyperthyroidism. Notably, increased mortality was associated with uncontrolled hyperthyroidism irrespective of therapy modality.

**Conclusions:**

Early radioiodine treatment will potentially reduce mortality and should be offered to patients with severe disease. However, thionamides are still suitable for patients with milder disease, contraindications to radioiodine, or individuals who choose to avoid permanent hypothyroidism. Ultimately, a patient individualised approach that prioritises early and sustained control of hyperthyroidism will improve long-term outcomes regardless of the therapy modality used.

## Background

The choice of primary therapy in Graves’ disease is one of the more contentious issues in clinical thyroidology. Three well established therapies, namely antithyroid drugs, radioiodine, and thyroidectomy have been available for decades, and their efficacy and limitations are well recognised (Table 1) [1, 2]. Thionamide drugs offer the prospect of remission without the need for lifelong thyroid hormone replacement but only about 45% of patients achieve long-term remission [3]. Ablative treatment with radioiodine or thyroidectomy result in better cure rates and overcome the risk of non-compliance with thionamides, but both treatments incur the need for permanent levothyroxine therapy [1, 2]. Thyroidectomy also carries risks of permanent hypoparathyroidism and laryngeal nerve damage while radioiodine may aggravate orbitopathy and entails exposure to radioisotopes which is unsuitable for young children, pregnant women, or patients planning pregnancy [1, 2]. In practical terms radioiodine has the added advantage that it is administered in a single outpatient visit without the risks and overheads of surgery.

**Table 1:** Pros and cons of Graves’ disease treatments

	Antithyroid drugs	Radioiodine	Thyroidectomy
**Pros**	• Prospect of euthyroid remission • Permanent hypothyroidism - rare • Used in pregnancy • Non-invasive	• Rapid control (weeks) • Cure rates ~ 80–90% • Single outpatient treatment • Side-effects minor and rare • Cost-effective	• Rapid control (days) • Cure rates ~ 100% for total thyroidectomy • Useful in patients with co-existent primary hyperparathyroidism, malignancy, large goitres or airway compression
**Cons**	• Low remission rates ~ 45% • Risk of relapse in future pregnancy or postpartum period • 12–18 months of treatment required • Major drug side effects: agranulocytosis, liver toxicity, cholestatic liver disease, ANCA positive vasculitis, acute pancreatitis • Risk of birth defects if used in first trimester of pregnancy	• Aggravation of orbitopathy • Permanent hypothyroidism • Radiation restrictions after treatment • Contraindicated in pregnancy due to risks of fetal anomalies and fetal hypothyroidism • Contraindicated in individuals imminently planning pregnancy • Need to wait 6 months after treatment before conception or fathering a child	• Anaesthetic risks • Risk of permanent hypoparathyroidism • Risk of recurrent laryngeal nerve damage • Permanent hypothyroidism • Neck scar • Best avoided in pregnancy due to surgical and anaesthetic risks on fetus and mother

Radioiodine is the preferred therapy in the United States whereas in Europe and most of Asia, it is reserved as second line for patients who relapse after initial thionamide treatment [4]. The recent guidelines of the UK National Institute for Health and Care Excellence (NICE) recommends that radioiodine should now be first line treatment for Graves’ disease in the UK (Table 2) [5]. This position represents a departure from current practice and is largely based on the superior cost-effectiveness and efficacy of radioiodine compared to thionamides [6]. The importance of treatment efficacy has also been highlighted by a number of recent observational studies that have shown an increase in long-term cardiovascular mortality in poorly controlled patients with hyperthyroidism [7–10]. However, a recent large-scale study in patients with hyperthyroidism reported increased cancer mortality in association with radioiodine treatment [11]. This study has raised concern regarding the safety of radioiodine and hence the implications of the NICE guidance. A critical appraisal of these and other treatment-related mortality studies in hyperthyroidism is therefore necessary to ascertain the safety of the proposed NICE approach.

**Table 2:** NICE guidelines on first line treatment for Graves’ disease in adults [5]

***1.6.10***	
Offer radioactive iodine as first-line definitive treatment for adults with Graves’ disease, unless antithyroid drugs are likely to achieve remission (see recommendation 1.6.11), or it is unsuitable (for example, there are concerns about compression, malignancy is suspected, they are pregnant or trying to become pregnant or father a child within the next 4 to 6 months, or they have active thyroid eye disease).	
***1.6.11***	
Offer a choice of antithyroid drugs (a 12 to 18-month course) or radioactiveiodine as first-line definitive treatment for adults with Graves’ disease if antithyroid drugs are likely to achieve remission (for example, mild and uncomplicated Graves’ disease).	
***1.6.12***	
Offer antithyroid drugs (a 12 to 18 month course) as first-line definitive treatment for adults with Graves’ disease if radioactive iodine and surgery are unsuitable.	

## Main text

Although the association between hyperthyroidism and adverse cardiovascular outcomes is well-recognised, only a handful of studies have specifically addressed the long-term consequences of treatment on mortality. Such outcomes are particularly relevant to Graves’ disease patients since they are relatively young and are expected to enjoy a lengthy survival in the aftermath of treatment. Furthermore, unlike patients with toxic nodules who require ablative treatment, Graves’ disease can be cured by all three treatments, making it essential to understand the lasting effects of treatment. There are no randomised controlled trials on long-term mortality outcomes in Graves’ disease and our understanding of treatment-related survival is based on data from observational studies conducted in a variety of settings and methodological designs. Our focus in this analysis is on studies that have evaluated total, cardiovascular, or cancer related mortality in radioiodine treated cohorts, either in comparison with the background population (Table 3) or with other treatment modalities (Table 4).

**Table 3:** Mortality studies in hyperthyroidism: treatment groups vs disease-free/population controls

Author (ref)	Year	Country	Setting	No of patients	Total/CV mortality RR (95%CI)	Cancer mortality RR (95%CI)
Radioactive Iodine vs disease-free/population controls						
Goldman [12] (a)	1988	USA	Hospital	1762	Increased; SMR 1.3 (1.2, 1.4)	No difference; SMR 0.9 (0.7, 1.1)
Goldman [12] (b)	1988	USA	Hospital	607	Increased; SMR 1.2 (1.1, 1.4)	No difference; SMR 1.0 (0.7, 1.3)
Hall [13, 14]	1992, 1993	Sweden	Hospital	10,552	Increased; SMR 1.5 (1.4, 1.5)	Increased; SMR 1.1 (1.0, 1.2)
Franklyn [15, 16]	1998, 1999	UK	Register	7209, 7417 (c)	Increased; SMR 1.1 (1.1, 1.2)	Decreased; SMR 0.9 (0.8, 0.9)
Ron [17]	1998	USA/UK	CTTFUS	35,593	NA	No difference; SMR 1.0 (0.9, 1.0)
Metso [18]	2007	Finland	Hospital	2793	Increased; SMR 1.1 (1.0, 1.2)	Increased; RR 1.3 (1.1, 1.6)
Kitahara [11]	2019	USA/UK	CTTFUS	18,805	NA	Increased; RR 1.1 (1.0, 1.1)
Antithyroid Drugs vs disease-free/population controls						
Ron [17]	1998	USA/UK	CTTFUS	35,593	NA	Increased; SMR 1.3 (1.1, 1.6)
Boelaert [7]	2013	UK	Hospital	1036 (d)	Increased; SMR 1.3 (1.1, 1.6) (d)	No difference; SMR 1.0 (0.7, 1.6)
Okosieme [8]	2019	UK	Registry	3587	Increased; RR 1.2 (1.0, 1.5)	NA
Thyroidectomy vs disease-free/population controls						
Ryodi [19]	2013	Finland	Registry	4334	No difference; RR 0.91, (0.8, 1.1) (e)	NA

***RR*** relative risks, ***CI*** confidence interval, ***CV*** cardiovascular, ***CTTFUS*** Cooperative Thyrotoxicosis Therapy Follow-up study cohort, ***SMR*** Standardised Mortality Ratio, ***NA*** Not assessed

**(a)**, 80% of cohort treated with RAI, **(b)**, Radioiodine treated cohort only, **(c)** Numbers are for the 1998 and 1999 cohorts respectively, **(d)** For reference [7] HR are based on person years accumulated during thionamide therapy in 1036 patients of which 272 received antithyroid drugs alone and 764 received radioiodine with or without antithyroid drugs, **(e)** RR for CV mortality

**Table 4:** Mortality studies in hyperthyroidism: treatment groups vs disease controls

Author (ref)	Year	Country	Setting	No of patients	Total/CV mortality RR (95%CI)	Cancer mortality RR (95%CI)
RAI vs Thyroidectomy						
Hoffman [20]	1982	USA	Hospital	1005 vs 2141 (RAI vs T)	No difference; RR 1.0 (0.9, 1.2)	No difference; RR 1.0 (0.7, 1.3) (a)
Ryodi [21, 22]	2015, 2018	Finland	Registry	1814 vs 4334 (RAI vs T)	Increased; HR 2.1 (1.7, 2.5) (b)	No difference; RR 1.0 (0.9, 1.2)
Giesecke [23]	2017	Sweden	Registry	10,250 vs 742 (RAI vs T)	Increased; HR 1.2 (1.0,1.4)	No difference; HR 0.96 (0.73, 1.3)
RA1 vs ATD						
Boelaert [7]	2013	UK	Hospital	764 vs 272 (RAI-Grp A vs ATD) **(7a)**	Reduced; HR 0.7 (0.5, 0.9)	NA
				764 vs 272 (RAI-Grp B vs ATD) **(7b)**	No difference; HR 0.9 (0.7, 1.3)	NA
Okosieme [8]	2019	UK	Registry	250 vs 3587 (RAI-Grp A vs ATD) **(8a)**	Reduced; HR 0·5 (0·3, 0.9)	NA
				182 vs 3587 (RAI-Grp B vs ATD) **(8b)**	No difference; HR 1·5, (0·9, 2·4)	NA
Gronich [24]	2020	UK	Database	2829 vs 13,808 (RAI vs ATD)	Reduced; HR 0.83 (0.72, 0.95)	No difference; HR 1.0 (0.8, 1.2) (a)

***RR*** relative risks, ***CI*** confidence interval, ***CV*** cardiovascular, ***RAI*** Radioactive Iodine, ***T*** Thyroidectomy, ***ATD*** Antithyroid Drugs, ***SMR*** Standardised Mortality Ratio

**(a)**, RR/HR for cancer incidence, **(b)**, HR for CV mortality. **(7a, 7b)** For reference [7], HR are based on person years accumulated after RAI and on Levothyroxine (RAI-Grp A) or person years after RAI but not taking or before taking Levothyroxine (RAI-Grp B) vs person years on thionamide alone (ATD). **(8a, 8b)** For reference [8], radioiodine groups were divided into patients with resolved hyperthyroidism after radioiodine (RAI-Grp A) and patients with unresolved hyperthyroidism after radioiodine (RAI-Grp B)

### Radioiodine vs population controls

Studies from the United States [12], Sweden [13], UK [15], and Finland [18] all reported increased all-cause mortality compared to the background general population in patients who received radioiodine therapy for hyperthyroidism (Table 3). In these studies, excess deaths were attributable to cardiovascular disorders, and in some cases, mortality increased with higher radioiodine doses [15]. A more varied picture is seen with cancer mortality, with reports of increased [11, 14, 18], similar [12] or even decreased [16], overall cancer mortality risk in radioiodine-treated patients with hyperthyroidism. In positive studies, increased cancer risk was dose dependent [11, 14, 18] and attributable to upper gastroesophageal [14, 18], respiratory tract [14] or breast tumours [11] suggesting that the malignancies were a consequence of internal exposure to radioactivity in iodide accumulating organs. Cancer mortality risk was also increased in younger versus older patients and in patients with toxic nodules compared to Graves’ disease patients [14]. Clearly a key limitation of these studies is the absence of hyperthyroid control groups that were not exposed to radioiodine thus making it difficult to distinguish the effects of radioiodine from those of hyperthyroidism per se. For the same reason, cumulative dose related risks cannot simply be ascribed to a radioiodine effect since patients with more severe disease would have received higher or multiple doses of radioiodine.

Of interest is the recent study by Kitahara et al which evaluated cancer mortality in 18,805 radioiodine-treated patients from the Cooperative Thyrotoxicosis Therapy Follow-up study cohort (CTTFUS) [11]. The CTTFUS is a long-running longitudinal cohort of more than 35,000 hyperthyroid patients enrolled between 1946 and 1964 in the United States and United Kingdom. Using a novel dosimetric model to estimate the tissue radioiodine dose, the authors showed a modest association between radioiodine treatment and solid cancer deaths (Relative Risk [RR] 1.06, 95% Confidence Interval [CI], 1.02–1.10). However, the study faces the same challenges in interpretation as other radioiodine cohort studies that lacked hyperthyroid control groups. In addition, the dosimetric model used in the study was based on a series of assumptions which resulted in uncertainties in the tissue dose estimates. Also, the study did not adjust for relevant confounders for cancer risk including smoking, obesity, alcohol intake, and thyroid status [11]. Lastly, the association with breast or solid cancer risk seems implausible given that the mean radioiodine dose in the study was < 500 MBq which is about a tenth of the usual treatment doses administered to thyroid cancer patients [25]. Solid tumours are not seen in association with the higher doses used in thyroid cancer treatment and only a small risk of leukaemia has been reported in thyroid cancer patients [25].

In an unusual development, two of the study co-authors have published a critique of their own study along with additional analysis from the same dataset but with the inclusion of hyperthyroid groups unexposed to radioiodine [26]. Using conventional standardised mortality ratios (SMR), this supplementary analysis showed no excess cancer mortality in radioiodine-treated patients but in contrast observed an increased cancer mortality in patients treated exclusively with antithyroid drugs (SMR 1.28, 95% CI 1.09–1.48) [26]. This mortality was due to deaths from cancers of the buccal cavity, digestive tract, and all solid cancers and are consistent with a previous analysis of the CTTFUS cohort by Ron et al which also used SMRs to show excess cancer deaths in thionamide treated but not in radioiodine-treated patients [17]. More recently, Gronich et al examined cancer risk in 16,637 hyperthyroid patients sourced from a health care database. Correcting for age, sex, smoking history and body mass index amongst other variables the authors found no association between radioiodine therapy and total or individual cancer risk in long-term follow up [24]. The reasons for these discrepancies are unclear and may reflect differences in analytical methods but nonetheless call for further clarification.

### Radioiodine vs thyroidectomy

A second approach has been to compare mortality outcomes in radioiodine versus thyroidectomy cohorts (Table 4). A 1982 Mayo clinic study showed no difference in total or cancer related mortality in radioiodine versus surgically treated women with hyperthyroidism [20]. Two recent national registry studies from Finland and Sweden have shown an increase in all-cause mortality [23] and cardiovascular mortality [21] in radioiodine treated patients compared to thyroidectomy patients. In contrast differences in cancer related mortality were not observed between the two groups in these cohorts [22, 23]. Again, these studies did not include thionamide only groups and did not account for the severity and duration of hyperthyroidism exposure before and after definitive treatment. In the Finnish study, excess cardiovascular morbidity was not seen in radioiodine treated patients who developed hypothyroidism or in surgically treated patients compared to the background population [19], suggesting that the survival advantages of surgery over radioiodine therapy may be related to the superior hyperthyroidism control achieved with thyroidectomy [21].

### Radioiodine versus thionamide

Three studies compared mortality in radioiodine versus thionamide treated patients (Tables 3 and 4). The first is the CTTFUS study by Ron et al which showed excess cancer deaths in thionamide but not radioiodine-treated patients [17]. A 2013 hospital clinic study by Boelaert et al comprising 1036 hyperthyroid patients compared mortality according to thionamide or radioiodine treatment [7]. Using a time-dependent regression model, they showed reduced mortality in association with radioiodine but only after it led to hypothyroidism, implying survival advantages of hyperthyroidism control. In a recent record linkage study in South Wales, UK, we evaluated cardiovascular morbidity and mortality according to the modality and effectiveness of primary therapy in Graves’ disease (*n* = 4189) [8]. We used a one-year landmark analytical approach to show that early and successful radioiodine treatment was associated with a 50% reduction in mortality compared to thionamide treatment but this survival advantage was lost when radioiodine failed to control hyperthyroidism by the one-year landmark. In addition the free T4 concentration after initial treatment was positively associated with long-term mortality and persistent hyperthyroidism carried a 55% increase in mortality risk independent of therapy modality [8].

Two recent Danish registry studies have also shown associations between cumulative hyperthyroidism exposure and mortality or cardiovascular events in patients with hyperthyroidism although treatment modality was not evaluated in these studies [9, 10]. The above findings support the well-established detrimental effects of thyroid hormone excess on the cardiovascular system, and this may explain some of the inconsistencies in previous data. In the study by Ron et al, increased cancer mortality in patients treated with antithyroid drugs can be explained by less effective thyroid control in thionamide treated patients rather than a carcinogenic effect of thionamides per se [17]. A similar mechanism may be at play for the superior outcomes observed for thyroidectomy compared to radioiodine [21–23] as well as the survival advantages of achieving a hypothyroid state in the aftermath of radioiodine therapy [21].

### Individualised treatment

Thus, in patients with hyperthyroidism, the risk of mortality, whether cardiocvascular or cancer mortality, appears to be driven by thyroid hormone excess above all*.* Even if there was a marginal risk of radioiodine related cancer, such risk would clearly be offset by the overall survival gains seen with disease control after radioiodine. The prospect of improving survival with better treatment has led some experts to suggest a ‘patient-tailored’ treatment approach to Graves’ disease management [27]. This strategy would offer early definitive therapy to patients with unfavourable characteristics such as severe disease, large goitres, or high TRAb levels, while thionamides are reserved for less severe cases. In this regard the current NICE guidelines are a step in the right direction and should enable more efficient individualised care (Table 2). However, the guidance risks being interpreted as an endorsement of radioiodine over other therapies whereas the reality is more nuanced. In particular the issue of therapy choice must not overshadow the central importance of controlling hyperthyroidism which is feasible with any therapy modality. In our study 39% of patients remained hyperthyroid one-year after diagnosis with an increased risk of subsequent death [8] underpinning the need for prompt control of hyperthyroidism as a core standard of care.

Thionamides are increasingly used as first line therapy for Graves’ disease globally, even in the United States where radioiodine has traditionally been preferred [4]. The extent to which this will change in the UK with the advent of NICE is uncertain since radioiodine would remain unsuitable for a sizable proportion of the Graves’ disease population including patients with orbitopathy, pregnant women, individuals intending pregnancy [28], or patients with young children. Some patients may prefer thionamides due to concerns with permanent hypothyroidism, weight gain, or reduced quality of life on levothyroxine. Such concerns are not unfounded. A study of Swedish Graves’ disease patients randomised to treatment with thionamides, radioiodine, or surgery, showed no difference in treatment satisfaction [29] or long-term quality of life between the treatment arms [30]. However, a more recent study, also from Sweden, in which Graves’ disease patients were followed up for 6–10 years after primary therapy, showed that the proportion of patients who felt fully recovered was significantly lower amongst those receiving levothyroxine compared to those not on levothyroxine [3].

In addition, some studies have suggested that weight gain after treatment of hyperthyroidism may be more pronounced with ablative therapy than antithyroid drug therapy [31]. A retrospective study in 133 hyperthyroid patients reported more weight gain in patients rendered hypothyroid with radioiodine or total thyroidectomy compared to those who remained euthyroid after thionamides or hemithyroidectomy [31]. In another study of 162 hyperthyroid patients an average weight gain of 5 kg and BMI increase of 8 kg/m^2^ was documented over a mean period of 24 months. Weight gain was similar for patients who received antithyroid drugs or radioiodine treatment but was significantly greater for patients who had been treated with thyroidectomy (5 kg vs 10 kg; *P* = 0.007). Overall, weight gain was associated with development of hypothyroidism despite levothyroxine replacement [32]. Thus, individuals who opt for thionamides should be reassured that their choices are valid and that optimal outcomes can still be achieved provided hyperthyroidism is controlled as early as possible.

Also relevant to treatment decisions are the resource and health economic implications of treatment. Thus, centres who do not have easy access to nuclear medicine facilities or lack the expertise of experienced high-volume thyroid surgeons may lean towards the use of antithyroid drugs over more definitive therapies. Such an approach is not unreasonable and centres should be equipped with robust protocols to use antithyroid drugs at doses and treatment durations that promote early and lasting resolution of hyperthyroidism. A cost-utility analysis by Donovan et al on the lifetime cost-effectiveness of the three first-line therapies in Graves’ disease in England and Australia, showed that radioiodine was more cost effective than antithyroid drugs or surgery but that antithyroid drugs still offered a cost-effective alternative to radioiodine [6]. However, these results were highly sensitive to the quality of life weights attached to post-ablative hypothyroidism and euthyroidism on antithyroid drug therapy.

## Conclusions

The controversy surrounding Graves’ disease management is likely to continue, but the emphasis must now move away from binary positions on treatment modality and seek pragmatic strategies to optimise the safety, efficiency, and outcomes of existing therapies. Regardless of therapy modality, a treatment approach that prioritises early control of hyperthyroidism will ultimately improve long-term outcomes. The challenge for clinicians will be to identify which patients will respond to thionamides and can be spared ablation and lifelong levothyroxine. A number of useful clinical scores for predicting thionamide response are in use [33, 34], but these scores will require further refinement and validation. Although randomised controlled trials with long-term mortality endpoints are now increasingly unlikely, further observational studies are needed to clarify the safety of radioiodine. Such studies will require thoughtful designs to avoid the usual traps of non-randomised data such as confounding by indication, lack of appropriate comparative groups, and bias from uneven treatment time exposures. Lastly, long-term quality of life studies will be essential to understand the impact of therapy on lifelong well-being.

## Data Availability

Not applicable

## References

[1] Kahaly GJ, Bartalena L, Hegedüs L, Leenhardt L, Poppe K, Pearce SH (2018). 2018 European thyroid association guideline for the management of Graves’ hyperthyroidism. Eur Thyroid J.

[2] Ross DS, Burch HB, Cooper DS, Greenlee MC, Laurberg P, Maia AL (2016). 2016 American thyroid association guidelines for diagnosis and management of hyperthyroidism and other causes of thyrotoxicosis. Thyroid..

[3] Sjolin G, Holmberg M, Torring O, Bystrom K, Khamisi S, de Laval D (2019). The long-term outcome of treatment for Graves’ hyperthyroidism. Thyroid.

[4] Taylor PN, Albrecht D, Scholz A, Gutierrez-Buey G, Lazarus JH, Dayan CM (2018). Global epidemiology of hyperthyroidism and hypothyroidism. Nat Rev Endocrinol.

[5] NICE (2019). Thyroid disease guidelines: National Institute of Health and Care Excellence.

[6] Donovan PJ, McLeod DS, Little R, Gordon L (2016). Cost-utility analysis comparing radioactive iodine, anti-thyroid drugs and total thyroidectomy for primary treatment of Graves’ disease. Eur J Endocrinol.

[7] Boelaert K, Maisonneuve P, Torlinska B, Franklyn JA (2013). Comparison of mortality in hyperthyroidism during periods of treatment with thionamides and after radioiodine. J Clin Endocrinol Metab.

[8] Okosieme OE, Taylor PN, Evans C, Thayer D, Chai A, Khan I (2019). Primary therapy of Graves’ disease and cardiovascular morbidity and mortality: a linked-record cohort study. Lancet Diabetes Endocrinol.

[9] Lillevang-Johansen M, Abrahamsen B, Jorgensen HL, Brix TH, Hegedus L (2017). Excess mortality in treated and untreated hyperthyroidism is related to cumulative periods of low serum TSH. J Clin Endocrinol Metab.

[10] Lillevang-Johansen M, Abrahamsen B, Jorgensen HL, Brix TH, Hegedus L (2019). Duration of hyperthyroidism and lack of sufficient treatment are associated with increased cardiovascular risk. Thyroid..

[11] Kitahara CM, Berrington de Gonzalez A, Bouville A, Brill AB, Doody MM, Melo DR (2019). Association of radioactive iodine treatment with cancer mortality in patients with hyperthyroidism. JAMA Intern Med.

[12] Goldman MB, Maloof F, Monson RR, Aschengrau A, Cooper DS, Ridgway EC (1988). Radioactive iodine therapy and breast cancer. A follow-up study of hyperthyroid women. Am J Epidemiol.

[13] Hall P, Lundell G, Holm LE (1993). Mortality in patients treated for hyperthyroidism with iodine-131. Acta Endocrinol.

[14] Hall P, Berg G, Bjelkengren G, Boice JD, Ericsson UB, Hallquist A (1992). Cancer mortality after iodine-131 therapy for hyperthyroidism. Int J Cancer.

[15] Franklyn JA, Maisonneuve P, Sheppard MC, Betteridge J, Boyle P (1998). Mortality after the treatment of hyperthyroidism with radioactive iodine. N Engl J Med.

[16] Franklyn JA, Maisonneuve P, Sheppard M, Betteridge J, Boyle P (1999). Cancer incidence and mortality after radioiodine treatment for hyperthyroidism: a population-based cohort study. Lancet..

[17] Ron E, Doody MM, Becker DV, Brill AB, Curtis RE, Goldman MB (1998). Cancer mortality following treatment for adult hyperthyroidism. Cooperative thyrotoxicosis therapy follow-up study group. Jama..

[18] Metso S, Jaatinen P, Huhtala H, Auvinen A, Oksala H, Salmi J. Increased cardiovascular and cancer mortality after radioiodine treatment for hyperthyroidism. J Clin Endocrinol Metab. 2007;92(6):2190–6. 10.1210/jc.2006-2321 17374710

[19] Ryödi Essi, Salmi Jorma, Jaatinen Pia, Huhtala Heini, Saaristo Rauni, Välimäki Matti, Auvinen Anssi, Metso Saara (2013). Cardiovascular morbidity and mortality in surgically treated hyperthyroidism - a nation-wide cohort study with a long-term follow-up. Clinical Endocrinology.

[20] HOFFMAN DANIEL A, MCCONAHEY WILLIAM M., DIAMOND EARL L., KURLAND LEONARD T (1982). MORTALITY IN WOMEN TREATED FOR HYPERTHYROIDISM. American Journal of Epidemiology.

[21] Ryödi Essi, Metso Saara, Huhtala Heini, Välimäki Matti, Auvinen Anssi, Jaatinen Pia (2018). Cardiovascular Morbidity and Mortality After Treatment of Hyperthyroidism with Either Radioactive Iodine or Thyroidectomy. Thyroid.

[22] Ryödi Essi, Metso Saara, Jaatinen Pia, Huhtala Heini, Saaristo Rauni, Välimäki Matti, Auvinen Anssi (2015). Cancer Incidence and Mortality in Patients Treated Either With RAI or Thyroidectomy for Hyperthyroidism. The Journal of Clinical Endocrinology & Metabolism.

[23] Giesecke P., Frykman V., Wallin G., Lönn S., Discacciati A., Törring O., Rosenqvist M. (2017). All-cause and cardiovascular mortality risk after surgeryversusradioiodine treatment for hyperthyroidism. British Journal of Surgery.

[24] Gronich Naomi, Lavi Idit, Rennert Gad, Saliba Walid (2020). Cancer Risk After Radioactive Iodine Treatment for Hyperthyroidism: A Cohort Study. Thyroid.

[25] Yu Chi Yun, Saeed Omar, Goldberg Alyse S., Farooq Shafaq, Fazelzad Rouhi, Goldstein David P., Tsang Richard W., Brierley James D., Ezzat Shereen, Thabane Lehana, Goldsmith Charlie H., Sawka Anna M. (2018). A Systematic Review and Meta-Analysis of Subsequent Malignant Neoplasm Risk After Radioactive Iodine Treatment of Thyroid Cancer. Thyroid.

[26] Tulchinsky Mark, Brill A. Bertrand (2019). Spotlight on the Association of Radioactive Iodine Treatment With Cancer Mortality in Patients With Hyperthyroidism is Keeping the Highest Risk From Antithyroid Drugs in the Blind Spot. Clinical Nuclear Medicine.

[27] Bartalena Luigi, Piantanida Eliana, Tanda Maria Laura (2019). Can a patient-tailored treatment approach for Graves' disease reduce mortality?. The Lancet Diabetes & Endocrinology.

[28] Okosieme Onyebuchi E., Khan Ishrat, Taylor Peter N. (2018). Preconception management of thyroid dysfunction. Clinical Endocrinology.

[29] Törring O, Tallstedt L, Wallin G, Lundell G, Ljunggren J G, Taube A, Sääf M, Hamberger B (1996). Graves' hyperthyroidism: treatment with antithyroid drugs, surgery, or radioiodine--a prospective, randomized study. Thyroid Study Group. The Journal of Clinical Endocrinology & Metabolism.

[30] Abraham-Nordling Mirna, Törring Ove, Hamberger Bertil, Lundell Göran, Tallstedt Leif, Calissendorff Jan, Wallin Göran (2005). Graves' Disease: A Long-Term Quality-of-Life Follow Up of Patients Randomized to Treatment with Antithyroid Drugs, Radioiodine, or Surgery. Thyroid.

[31] Kyriacou A, Kyriacou A, Makris KC, Syed AA, Perros P. Weight gain following treatment of hyperthyroidism-a forgotten tale. Clin Obes. 2019;9(5):e12328. 10.1111/cob.12328 31267667

[32] Dale J., Daykin J., Holder R., Sheppard M. C., Franklyn J. A. (2001). Weight gain following treatment of hyperthyroidism. Clinical Endocrinology.

[33] Vos Xander G., Endert Erik, Zwinderman A. H., Tijssen Jan G. P., Wiersinga Wilmar M. (2016). Predicting the Risk of Recurrence Before the Start of Antithyroid Drug Therapy in Patients With Graves' Hyperthyroidism. The Journal of Clinical Endocrinology & Metabolism.

[34] Masiello E., Veronesi G., Gallo D., Premoli P., Bianconi E., Rosetti S., Cusini C., Sabatino J., Ippolito S., Piantanida E., Tanda M. L., Chiovato L., Wiersinga W. M., Bartalena L. (2018). Antithyroid drug treatment for Graves’ disease: baseline predictive models of relapse after treatment for a patient-tailored management. Journal of Endocrinological Investigation.

